# Dipolar colloids in three dimensions: non-equilibrium structure and re-entrant dynamics†

**DOI:** 10.1039/d5sm00182j

**Published:** 2025-05-20

**Authors:** Nariaki Sakaï, Katherine Skipper, Fergus J. Moore, John Russo, C. Patrick Royall

**Affiliations:** a Institut Langevin, ESPCI Paris, Université PSL 75005 Paris France; b H. H. Wills Physics Laboratory, University of Bristol Bristol BS8 1TL UK; c Department of Physics, Sapienza University of Rome P.le Aldo Moro 5 00185 Rome Italy; d Gulliver UMR CNRS 7083, ESPCI Paris, Université PSL 75005 Paris France paddy.royall@espci.psl.eu

## Abstract

Understanding of collective behaviour in active systems is massively enhanced by minimal models which nevertheless capture its essence. Active colloids, whose interactions can be tuned and accurately quantified provide a valuable realisation of suitable basic models in an experimental setting and may even mimic certain biological systems. Experimental work on active colloids is dominated by (quasi) two-dimensional systems, but rather less is known of 3D systems. Here we investigate a 3D experimental system of active colloids up to volume fractions of 0.5. The particles in our system are self-propelled in the lateral plane under an AC electric field using induced-charge electrophoresis. The field in addition induces an electric dipole, and the competition between activity and both steric and dipolar interactions gives rise to phase behaviour ranging from an active gas to a dynamic labyrinthine phase as well as tetragonal and hexagonal crystals at a high volume fraction. Intermediate volume fractions are characterised by two-dimensional sheets with large fluctuations reminiscent of active membranes. These active sheets break symmetry in a direction perpendicular to the applied field. Moreover, the relationship between electric field and the particle dynamics depends in a complex and unexpected manner upon the position in the state diagram.

## Abstract 2

Colloids provide a unique platform in which to explore the effect of activity upon well-controlled thermal many-body systems. Until now, experiments have been largely limited to 2d. This work describes the phase behaviour and dynamics of a 3d active colloidal system.

## Graphical abstract

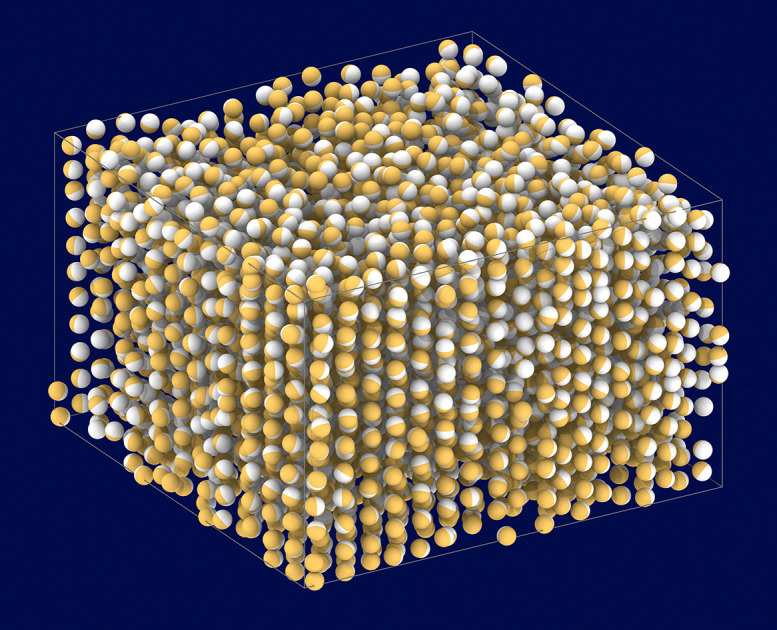

## 1 Introduction

Colloidal systems have a two-fold importance. They constitute important materials in their own right, from colloidal crystalline opals to cosmetics, foods, pharmaceuticals and agrichemicals. They are also prized for their tuneable interactions which enable simple and well-understood theoretical models to be rigorously compared with experiment.^1–3^ It is in this context as well-controlled model systems that active colloids^4^ have provided important insight into collective behaviour,^5–7^ such as flocking and swarming^8,9^ that is exhibited by biological systems such as bacteria,^9,10^ biological tissues,^11,12^ actin filaments^13,14^ midges^15^ and birds.^16^

Underlying much of the interest in (passive) colloids is their self-assembly to form a very wide range of ordered and crystalline structures.^17^ The combination of colloidal self-assembly and activity might thus be expected to yield exciting possibilities for new structures and new materials.^18–20^ Results from computer simulation, for example enhanced crystallisation^21^ emphasise the potential for this combination of colloidal self-assembly and activity. Experiments find living crystals,^22^ clustering,^23,24^ banding^25^ self-assembled microgears^26^ and novel dynamics^27^ while the exploitation of non-reciprocal interactions reveals even more exotic features such as odd elasticity.^28–30^ Among the exciting aspects of active colloids is their use of external electric^4,25,31^ and magnetic^4^ fields to readily tune the behaviour of the system.

As impressive as these achievements undoubtedly are, an important step forward to achieving the ambitious goal of active colloidal self-assembly in experiments is to develop 3D active colloidal systems, which forms the subject of this work. Predictions from computer simulation include motility-induced phase separation at high activity,^32–34^ and strong dynamic heterogeneity under pinning^35^ and novel polymorphic behaviour.^36^

As for our 3D active colloidal system, we implement the induced-charge electrophoresis mechanism, which exploits a Janus particle architecture of a metal cap on one hemisphere of a dielectric particle in an AC electric field.^37–39^ The 2D version of this system has been studied,^38–40^ in which dipolar interactions are induced by the electric field in both the metallic and dielectric parts of the particle. The magnitude and even the sign of the dipoles can be controlled with the frequency of the AC field, which enables a considerable degree of control over the assembly.^40,41^ For some frequencies in the field, both dipoles have a similar strength and magnitude, which we exploit here. In our 3D system the motility is in the lateral *xy* plane and the dipolar interaction lies in the *z* direction.

What can we expect from our 3D active dipolar colloids? By referring to computer simulation work^18,42^ a natural starting point is the passive parent system *i.e.* with the same direct interactions, but without activity.^18^ Dipolar colloids have been investigated extensively and exhibit a rich phase behaviour including a fluid, string fluid, and hexagonal close packed, body-centered cubic, body-centred orthorhombic and body-centred tertragonal (bct) crystalline structures.^43–45^ This sets a framework for the kind of self-assembly which may be influenced by the interplay of activity and direct interactions.

By analogy then, we might expect the phase behaviour we encounter to be similar to a passive system, albeit with the possibility of some active phenomena *e.g.* motility-induced phase separation (MIPS) that computer simulations and theory showed to also occur in three dimensions.^32,46–48^ Compared to such expectations, our system presents three unexpected findings. Firstly, the sheet phase observed only transiently with passive dipolar colloids.^49^ Here our active system forms a non-equilibrium steady state where sheets branch continuously and regenerate leading to an evolving structure.

Secondly, upon further increase of the volume fraction, the sheets percolate to form a dynamic labyrinth state. Here the symmetry is broken along the field direction (in which the structure is replicated) while in the plane perpendicular to the field it is an active network which branches continuously and regenerates leading to an evolving structure. Thirdly, contrary to expectations, we find re-entrant dynamical behaviour in that the relaxation time of our system decreases upon crystallisation.

This article is organised as follows. In our methodology (Section 2), we describe our experiments in Section 2.1 and the bond order parameters that we use to distinguish the sheet, labyrinth and the hexagonal and tetragonal crystal phases that we find in Section 2.2. Our results (Section 3) is divided into the phase behaviour (Section 3.1), characterisation of the structures found (Section 3.2), the emergence of the dynamic labyrinth (Section 3.3) and the re-entrant dynamics in Section 3.4. We discuss our findings in Section 4 and conclude in Section 5.

## 2 Methodology

### 2.1 Experimental

**Fig. 1:**
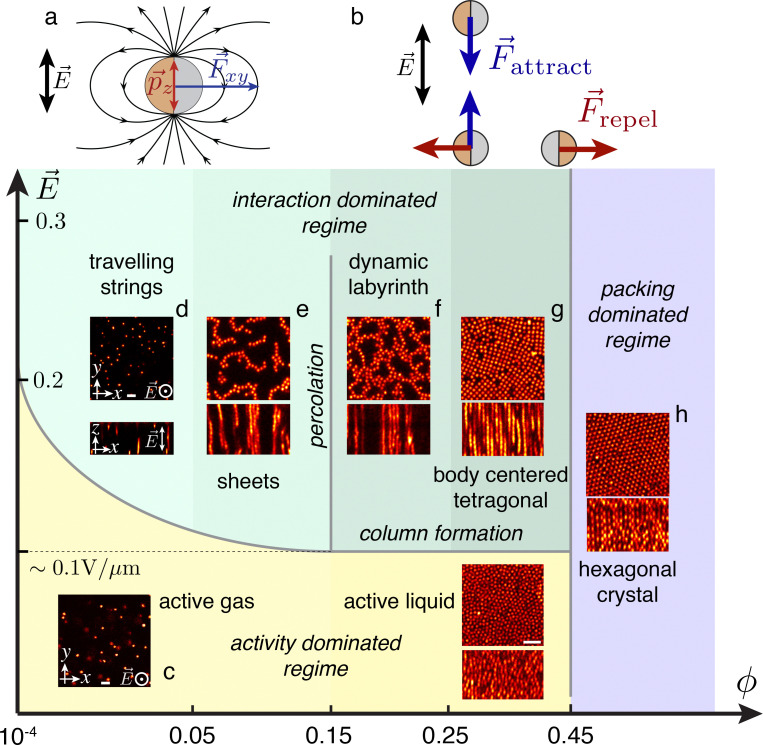
Phase diagram for induced-charge electrophoresis particles in 3D. (a) The external AC electric field *E⃑* induces an active force *F⃑*_*xy*_ and electric dipole *p⃑*_*z*_. The latter leads to dipolar interactions between the particles. (b) This interaction is anisotropic: two particles aligned with the electric field attract each other, whereas when they are perpendicular, the interaction is repulsive. There is a competition between the dipolar interaction and activity, which leads to a complex phase behaviour observed in our experiments. For our parameters, the metallic hemisphere trails behind the dielectric hemisphere.^40^ (c) At low field strength, the dipolar interaction is negligible and the suspension is in an active fluid state. Increasing the field leads to self-assembly into 1D strings (d) 2D sheets (e) and 3D labyrinthine phase (f) or tetragonal crystal (g), depending on the volume fraction, and these states exhibit possess astonishing dynamical behaviour. At the highest volume fraction, packing dominates; the suspension crystallises and adopts a hexagonal structure whatever the electric field (h). Images in the state diagram are two dimensional slices of 3D images obtained from our confocal microscope. Scale bars are 10 μm throughout the figure.

The active colloids are self-propelled by induced-charge electrophoresis.^39,40^ The net active force is parallel to the electrodes [Fig. 1(a)]. Consequently, isolated particles move similarly to active Brownian particles in a plane orthogonal to the field (*xy*) and (only) diffuse in the third direction (*z*). Moreover the resulting interactions between the particles can be modelled by two effective electric dipoles, one in each hemisphere of different magnitudes, reflecting the different materials from which the two hemispheres of the Janus particles are comprised.^50^ The dipolar interactions in this system have been carefully characterised and have a complex dependence on the frequency of the applied electric field.^40^ For our parameters (5 kHz and NaCl at a concentration of 10^−4^ mol L^−1^), both dipoles have similar strengths, and so for simplicity we treat our particles as a single effective dipole located at the centre. Thus the interaction between two particles depends on their orientation: they attract when aligned with the field, and repel if orthogonal [Fig. 1(b)].

We used two experimental systems depending on the volume fraction under consideration. In our first system, which we used for higher and intermediate volume fractions, the colloids have diameter *σ* = 1.5 μm silica particles labeled with rhodamine dye (Kisker Biotech), one hemisphere of which is covered with 3 nm of chromium using a thermal evaporator, then by another 15 nm shell of silica.^51^ The thickness of the metal is chosen such that the layer remains transparent. The particles are suspended in a mixture of milliQ water and DMSO at volume ratio 7 : 10 for refractive index matching. In this solvent mixture, the Brownian time to diffuse a diameter *τ*_B_ = 3.06 s. This system is not density matched between colloids and solvent, such that the gravitational length *l*_g_ ≈ 0.15*σ* and is therefore more suitable for volume fractions *ϕ* ≳ 0.1 where refractive index matching is important, but sedimentation is suppressed by the activity of the system.

At low volume fraction, the strings sediment markedly,^52^ so here we use a second system for *ϕ* less than 0.05. This consists of 1 μm fluorescent polystyrene particles also labeled with rhodamine (Invitrogen). These are covered with 5 nm of aluminium, then 5 nm of silica. The solvent is a mixture of water and glycerol at volume ratio 1 : 1, which allows us to density match the particles with the solvent and increase the viscosity. This system is not refractive index matched between colloids and solvent, limiting its use at higher volume fraction. It is furthermore hard to track the particle coordinates in this system.

The suspension is loaded into a cell formed by two ITO cover slips (SPI Supplies) spaced by a distance *h* ≈ 40 mm, and an AC electric field is applied (Black star Jupiter 2010) at a frequency of 5 kHz (unless otherwise indicated) at different voltage. The system is imaged using a Leica TCS SP8 confocal microscope, and particles are tracked in space using ref. 53, and trajectories are reconstructed following.^54,55^ The confocal microscope can acquire a 3D image in around 1s. This is sufficient to track slow-moving particles using the methods in ref. 54 and 55. Here our quantitative dynamical analysis is carried out on such data. In ref. 52, details of tracking the string phase can be found. For samples with polystyrene particles, the lack of index matching does not allow us to obtain particle trajectories, but we measured average velocity by manually recording individual displacements of ten particles between two successive frames. Where time-averaged data is shown (for the first system, with silica particles), we allowed the system at least 5 minutes (≈100*τ*_B_) to reach a steady state.

### 2.2 Local structural order parameters

To quantify the formation of vertical structures, we proceed as follows. For the *i*th particle, we set1
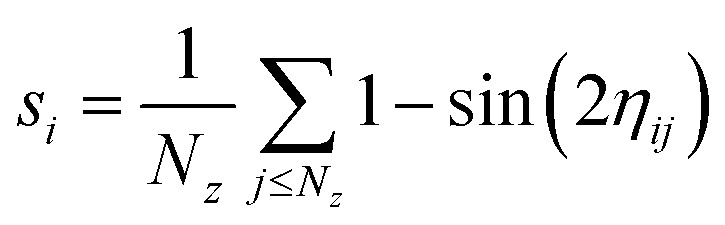
Here *η*_*ij*_ is the inclination angle between the bond formed by central particle *i* and neighbouring particle *j* with the *z* axis, and the sum is taken over the neighbours *N*_*z*_(*i*) composed of all the particles at a distance less than 1.5*σ* with a bond formed where the particle *i* has an inclination *i.e. η*_*ij*_ < π/4. *s*_*i*_ reaches its maximum at 1 for *η*_*ij*_ = 0 *i.e.* the particles *i* and *j* are aligned with the field, and is bounded below by 0 reached when *θ*_*ij*_ = π/4.

To characterise the tetragonal and hexagonal crystalline structures, we combine methods inspired from hexatic order parameters and first neighbour correlation.^56,57^ First, the tetragonal structure is characterized by computing for each particle *k* the quasi-2D 4-bond orientational order parameter:2
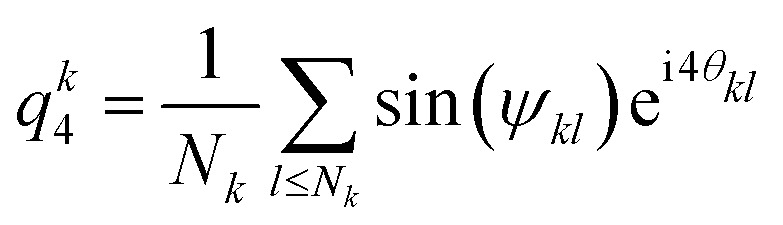
where *θ*_*kl*_ and *ψ*_*kl*_ are respectively the polar and azimuthal angle of the vector connecting particle *k* and the neighbouring particle *l*, and where the sum runs over all first neighbours *N*_*k*_. We used a sine weight to remove the contributions of top and bottom particles, which are always present as long as strings form. Then, for each particle *k*, we compute the neighbouring correlation3
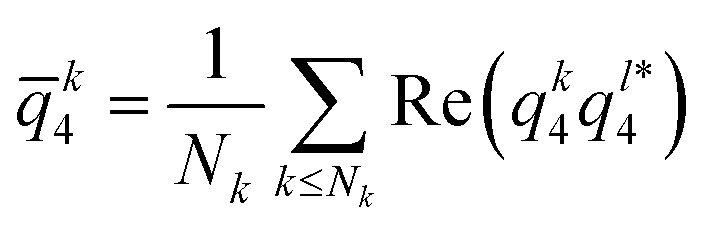
which corresponds to the average scalar products of the bond orientational order parameter of particle *k* with its neighbouring particles. This quantity ranges from −1 to 1, and locally crystallised particles – in a cubic lattice – have values located around unity, in contrast with those which are not in a cubic lattice where the values are located around zero.^57^

For the hexagonal structure, similarly to the tetragonal case, we compute the quasi-2D bond order parameter:4
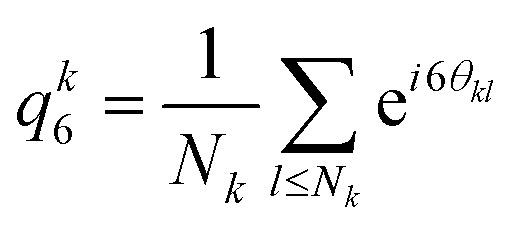
where this time, the sum runs over all neighbouring particles which are in the same *xy* plane *i.e.* has the same *z* coordinates modulo half diameter of particle. Then we compute the correlation *q̄*^*k*^_6_ using the same expression as for *q̄*^*k*^_4_.

## 3 Results

### 3.1 Phase behaviour

Consistent with expectations, at low volume fraction, we find a 3D “active gas”, where the activity is in the *xy* plane (Fig. 1(b) and Supplementary Movie 1, ESI†). Here for times which are short relative to the rotational diffusion time (4.1 s), the velocity is approximately measured by taking the displacement between two successive frames to be ∼100 μm s^−1^. When the dipolar interactions exceed the thermal energy, strings form and if the number density of particles is sufficient chains span the entire height of the sample (Fig. 1(c)). The symmetry is thus broken in the sense that the *xy* plane presents a variety of different phases (depending on field strength and colloid volume fraction), whereas the profile in the vertical direction is rather uniform. These strings then interact with each other depending on their number density. At very low density, the separation is large enough so that strings barely interact. These strings then travel in the *xy* plane, where their average velocity is ∼1 mm s^−1^ at a field strength of 0.3 V mm^−1^, presumably due to the un-aligned orientation of the particles in the strings so that there is some cancellation of the active forces, and which is 2 orders of magnitude smaller than for individual particles as can be estimated by linear extrapolation from data in ref. 40 (Movie S2, ESI†). The behaviour of the strings is further explored in ref. 52.

When the volume fraction is increased above *ϕ* ≈ 10^−2^, the interaction between the strings leads to self-organisation into sheets (Fig. 1(d)). In our system, these are active and exhibit a remarkable dynamical behaviour with strong fluctuations in the *xy* plane (see Movie S3, ESI†). When the volume fraction reaches around *ϕ* ≈ 0.15, we find that the active sheets percolate. The resulting structure is reminiscent of a labyrinth (Fig. 1(e)), but, as shown in Movie S4 (ESI†), the system is dynamic and reorganises over time, with opening and closing pathways through the labyrinth (see Movie S5, ESI†). At higher volume fraction still (*ϕ* ≈ 0.3), we find an assembly into a body-centered tetragonal (bct) crystal (see Movie S6, ESI†), and above *ϕ* ≈ 0.45 to a crystal with local hexagonal symmetry which is a mixture of FCC and HCP (see Movie S7, ESI†). This crystallisation is reminiscent of passive systems with similar dipolar interactions.^43,56^

Some comments on the sharpness of the phase boundaries are in order. Phase transitions in active matter are not yet fully understood, although some evidence for nucleation-like behaviour has been obtained.^33,58^ In passive dipolar colloids, the development of strings is known to be continuous.^59,60^ We shall see below that here, the strings form quite abruptly as a function of field strength. Although at the frequency that we have been able to sample state space, the phase boundaries seem reasonably sharp, and there seems no reason not to expect a continuous transition with respect to the field strength. We return to this point in the discussion.

### 3.2 Structural characterisation: order parameters for string, sheet, labyrinth and crystal structures

**Fig. 2:**
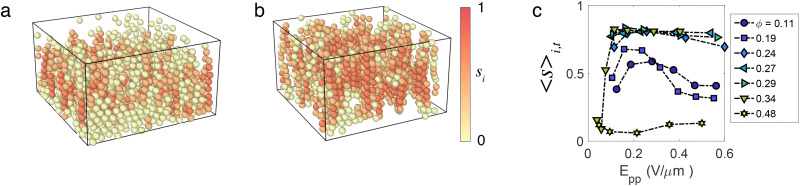
Sheet structures. (a) and (b) 3D rendered snapshots of the sheet state (*ϕ* = 0.1) for *E*_pp_ = 0.13 V mm^−1^ (a) and *E*_pp_ = 0.28 V mm^−1^. (b) Particles are coloured according to the value of their string order parameter 〈*S*_*i*,*t*_〉_*i*,*t*_. The average is taken over all particles *i* and time. (c) Mean string order parameter 〈*S*_*i*,*t*_〉_*i*,*t*_ with respect to field strength *E*_pp_ and volume fraction *ϕ*.

To characterise the different phases we consider the order parameter 〈*s*〉 (eqn (1)). Fig. 2(a) and (b) show 3D rendered snapshots of the sheet phase at two different field strengths; where particles are coloured according to their value of *s*_*i*_. For low field strength where sheets have barely formed [Fig. 2(a)], only a few particles have a non-zero value of *s*_*i*_, whereas at higher field strength [Fig. 2(b)], one observes well-defined string-like structures having *s*_*i*_ close to unity.

Here we see that 〈*s*〉 retains a large value up to *ϕ* = 0.34. At higher volume fraction, he local crystal symmetry acts to reduce 〈*s*〉. For the sheet, labyrinth and bct states 〈*s*〉_*i*,*t*_ takes a low value of ≈0.1 for *E*_pp_ < 0.1 V mm^−1^, and rapidly increases for higher fields before reaching a roughly stationary value which depends on the phase. This suggests that strings form at *E*_pp_ ≈ 0.1 V mm^−1^. For the sheet (*ϕ* = 0.11) and labyrinth (*ϕ* = 0.19) phases, the stationary value is located around 〈*s*〉_*i*,*t*_ ≈ 0.5, and drops at higher field strength (above *E*_pp_ ≈ 0.2 V mm^−1^). This suggests that either higher activity and/or stronger dipolar intercations influence the structure of the sheets/labyrinth. For the bct crystal, the stationary phase is located at 〈*s*〉_*i*,*t*_ ≈ 0.8, which means that most of the particles formed strings. In contrast with these three states, the hexagonal phase has a low 〈*s*〉_*i*,*t*_ whatever the electric field, meaning that strings barely formed in this phase.

Our second method to characterise the system is bond-orientational order (BOO) parameters. In particular, *q̄*^i^_4_, *q̄*^i^_6_ defined for each particle *i*, and based on quasi-2D bond orientational order parameter and first neighbour correlation^56,57^ (see the ESI†). These quantities are constructed such that they range from −1 to 1. Particles in a locally disordered structure have *q̄*_4_ = 0, *q̄*_6_ = 0, whereas particles in a tetragonal or hexagonal structure have *q̄*_4,6_ = 1.^57^ We can thus attribute to each particle a local structure by thresholding: particles having *q̄*_4_ > 0.5 and *q̄*_4_ > *q̄*_6_ are taken to be in a tetragonal structure, whereas *q̄*_6_ > 0.5 and *q̄*_6_ > *q̄*_4_ are in a hexagonal structure.

**Fig. 3:**
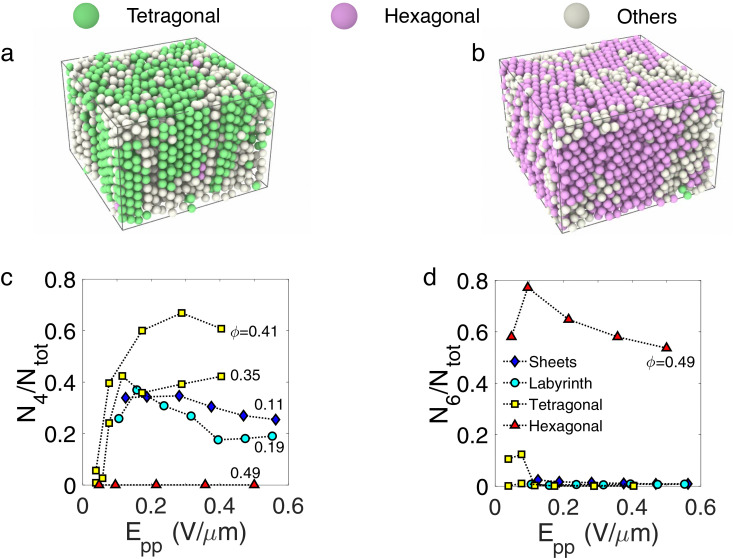
Crystalline polymorphs—(a) and (b) 3D rendered images obtained from tracking results of respectively tetragonal and hexagonal phases. Colours correspond to the local crystalline order obtained from *q̄*_4_ and *q̄*_6_ as explained in the text. (c) Time averaged crystal populations expressed as the fraction of particles in a bct crystal environment *N*_4_/*N*_tot_ as a function of field strength at the volume fractions indicated. (d) Time averaged crystal populations expressed as the fraction of particles in a hexagonal crystal environment *N*_6_/*N*_tot_ as a function of field strength at the volume fractions indicated. The phases in both (c) and (d) are indicated in the legend in (d). Lines are labelled with the volume fraction at which the data were taken.

Fig. 3(a) and (b) shows rendered 3D images from the sheet to the hexagonal phases where particles are coloured according to the local crystal structure, and show crystal domains in the tetragonal and hexagonal phases. We see that the sheet and labyrinth phases have a non-negligible number of particles in a tetragonal structure of about 35%. Moreover, some particles also have a local hexagonal structure, and the hexagonal phase also has a few particles identified in a tetragonal structure. To quantify the dependence upon the density and the electric field, we define the crystal population *N*_4_/*N*_tot_, *N*_6_/*N*_tot_ as the proportion of particles in a local tetragonal and hexagonal structure respectively. These quantities are plotted in Fig. 3(c) and (d), and show that the tetragonal crystal population increases with the electric field from zero to reach a stationary value at *E*_pp_ ≈ 0.1 V mm^−1^, while the hexagonal crystal population is non-zero and is almost independent of the electric field. Interestingly, strings in phases at lower volume fraction (string, sheet and labyrinth phases) also appears above *E*_pp_ ≈ 0.1 V mm^−1^. All these suggest that, similarly to the passive case,^43^ the hexagonal structure is governed by packing in contrast with the tetragonal structure that is determined by dipolar energy.

### 3.3 Emergence of the dynamic labyrinth

**Fig. 4:**
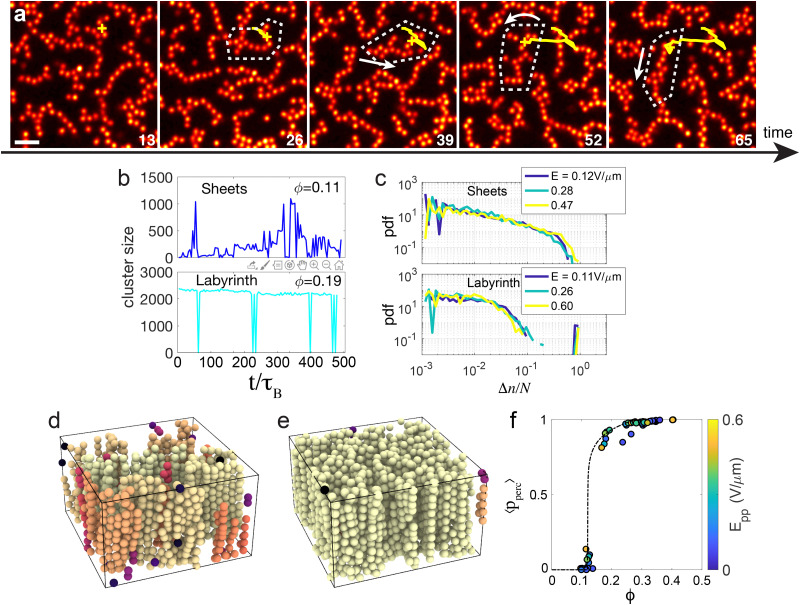
Emergence of the dynamic labyrinth—(a) 2D *xy* slices from confocal images under *E*_pp_ = 0.26 V μm^−1^. Here reorganisation resulting from local displacements is indicated. The scale bar corresponds to 5 microns. (b) Time-evolution of numbers of particles in the largest cluster in the image for sheet and dynamic labyrinth as indicated. (c) Probability distribution of size fluctuations Δ*n* between frames. (d) and (e) 3D rendered image where each particle is coloured depending on the size of the aggregate of connected particles it belongs to, at (d) *ϕ* = 0.11 and (e) 0.19. (f) Percolation order parameter *p*_perc_ (defined in the text) with respect to volume fraction. Colour of the symbols correspond to the electric field applied to the sample. Line is a guide to the eye.

The active sheet state is characterised by a large fluctuating dynamical behaviour with breaking and reforming of links between sheets. Such events are indicated in Fig. 4(a), where an assembly of particles detaches from a sheet, travels, and sticks to another sheet. That is to say, particles detach from one sheet and reattach to one another so that the size of the sheet they belong to changes all the time. Indeed, in Fig. 4(b), we display the time evolution of the size of the cluster that one arbitrary particle belongs to. We emphasise that this is an extensive quantity, characterising the local connectivity of the particles. Here a sheet is defined as an assembly of particles connected together with a bond length of 1.2*σ*, which is the first minimum of the pair correlation function. We can see that in the sheet phase, the size changes continuously, jumping from a very low value to a value close to the total number of particles. This results from a population of unattached particles which periodically attach to the cluster which comprises the vast majority of particles in the system. In contrast, in the labyrinthine phase, the cluster size is close to the total number of particles in the volume sampled except occasionally dropping to one, indicating a rare jump event occurring.

Fig. 4(c) quantifies the variation of cluster sizes in both sheet and labyrinthine states. In particular, we define the number of particles in the cluster *n* and the probability of observing a variation of size Δ*n* between two successive frames, normalised by the total number of particles tracked in this dataset *N*. For sheets [Fig. 4(c)], cluster size variations at all scales up to 1 are present, whereas for the labyrinthine state, most of the size variations are less than 5% of the total number of particles present in the observation window of the microscope, and there is a local maximum at Δ*s*/*n* = 1 as expected from Fig. 4(b). Interestingly, the dynamics of jumps does not seem to depend on the electric field.

Overall, these changes in dynamical behaviour are related to a percolation transition occurring in the *xy* plane. Fig. 4(d) and (e) shows two rendered 3D images of the system at a volume fraction of *ϕ* = 0.11 and *ϕ* = 0.19 respectively, where particles are coloured depending on the percolating cluster they belong to. At *ϕ* = 0.11, the sheets span in the direction parallel to the electric field but are separated in the *xy* plane whereas at *ϕ* = 0.19, the system percolates (at least on the length scale of our images). The change in the structure can be captured using the standard order parameter for percolation *i.e.* the probability *p*_perc_ that a given particle belongs to the “infinite” cluster, where we define the infinite cluster as the largest cluster that spans the sample and connects the six boundaries of the 3D image. This quantity is averaged in time, and is plotted in Fig. 4(f). The time averaged 〈*p*_perc_〉 is zero for densities less than 0.15 and non-zero for larger densities. Moreover, there is a clear discontinuity in the slope of the order parameter at 0.15, which is zero below 0.15 and close to infinity above, which is a signature of a percolation transition. Now the region sampled in our images is not large enough to determine precisely the percolation volume fraction. Therefore, here we have an estimate of the volume fraction at which percolation occurs in our system. Of course, the dynamic labyrinth requires activity. We find that, for field strengths greater than ≈0.1 V μm^−1^, the labyrinth and sheet phase are found, while at lower field strength we encounter an active liquid (see Fig. 1).

### 3.4 Particle transport and reentrant dynamics

**Fig. 5:**
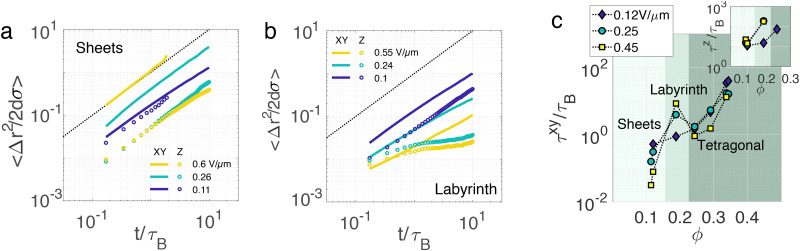
Diffusive dynamics and particle transport—(a) and (b) Mean square displacements (MSD) in *xy* (orthogonal) and *z* (parallel) directions with respect to the field. Data for sheet (*ϕ* = 0.11) and labyrinth (*ϕ* = 0.21) structures are shown in (a) and (b) respectively. Here *xy* data are indicated with a solid line and *z* data with data points. (c) Measurements of the relaxation time – in units of the Brownian time *τ*_B_ – with respect to volume fraction at low, middle and large electric fields. The main panel corresponds to the relaxation time measured from the MSD in the *xy* plane and the inset corresponds to displacements in the *z* direction. Here the three background colours represent the three phases.

Interestingly, this complex phase behaviour leads to markedly different transport regimes depending on the phase the system is in. Fig. 5(a) and (b) show the mean square displacement for the active sheets and labyrinth respectively. First, the mean square displacement is larger in the *xy* plane than in the *z*-direction, which is consistent with the fact that activity is directed in the *xy* plane. Second, the active sheet phase exhibits a diffusive behaviour both in the *xy* plane and the *z* direction, whereas it becomes subdiffusive for the labyrinthine state and the crystalline phases. Indeed, when the system percolates, most of the particles are bound. It is interesting to see that even in the tetragonal crystal, particles still diffuse at rather short timescales. Third, and importantly, the particle transport increases or decreases with the field depending on the phase of the system: the sheets and the crystal shows an increase of the transport with the electric field, while the labyrinth shows the opposite. This can be explicitly quantified by computing the self-intermediate scattering function *F*(*k*,*t*) and measuring the structural relaxation time *τ*, which for a suitable choice of wave-vector (*k* = *σ*/(2π)) quantifies the time needed for one particle to move a distance on the length scale of one diameter. Here we determine the relaxation time *τ* with a stretched exponential fit.

Fig. 5(c) shows the relaxation time for particle transport in *xy* and *z* respectively. First and generally speaking, the relaxation times increase with the volume fraction. Then, in the *xy* plane [Fig. 5(c)], while the sheet phase exhibits a substantial decrease of the relaxation time *i.e.* an acceleration of the dynamics with the field, the labyrinth features a decrease of the dynamics of the same order of magnitude. Then, in the tetragonal phase we see again an acceleration of the dynamics with the field. For the dynamics in the *z* direction [Fig. 5(c) inset], we can only measure the relaxation time at a relatively low density where the relaxation takes place in a range of time accessible to the experiments. In these cases, the relaxation time always increases with the electric field, except the sheet phase which is rather insensitive to the field strength.

## 4 Discussion

The 3D active system that we have realised exhibits a rich phase behaviour with a number of unexpected features based on predictions from computer simulations and expectations based on active colloidal systems, in 3D^32,46–48^ and passive dipolar colloids.^43,56,60^ Firstly, in contrast with the passive case,^49^ our sheets and labyrinth are stable. We believe that this is due to activity which brings large fluctuations and acts to suppress relaxation to a (condensed) bct crystal-string fluid phase coexistence. To our knowledge, it is rather unusual that activity can stabilise an ordered structure which is unstable in the passive case, although we note that in simulations of active Lennard-Jones particles, percolating network structures form steady states, rather than condensing case would be the case for a passive system.‡‡While of course phenomena such as motility-induced phase separation correspond in a sense to a structuring which requires activity, here our interest lies in ordering at the microscopic particle-level rather than larger-scale demixing, though we note that activity can drive crystallisation at very high volume fraction.^33^ ^42^ This sheet phase then undergoes a 2D percolation transition to a labyrinthine phase, which is only observed transiently in the passive parent system^49^ unlike the non-equilibrium steady state that we encounter here. A coupling of hydrodynamic interactions and activity lead the stabilisation of the sheet and labyrinth phases. How exactly activity may stabilise the labyrinth and sheet phases is an intriguing question. In non-equilibrium passive systems, it is known that hydrodynamic interactions can suppress condensation.^61,62^ We speculate that that a coupling of hydrodynamic interactions and activity might similarly suppress the condensation to a bct-fluid phase coexistence. We propose that in the future this may be investigated using computer simulation including hydrodynamics, as has recently been done for (quasi-) 2D active colloids.^63^ Despite the differences from the passive parent system, it seems that the dipolar interactions underlie much of the structure and phase behaviour that we see. At low volume fraction, we see strings and at higher volume fraction bct and hexagonal crystals. However, these transitions likely do not occur at the same state points here as they do in the passive case,^44^ which could be probed in detail using computer simulation. We also see that this is a suitable system to investigate the nature of phase transitions in active matter. It is quite possible that some of these phase boundaries may be first-order-like, and that some (like the string formation) would be continuous. Again, in addition to further experiment, this could be studied using computer simulation in order to access state points with higher precision than may be done using experiments.^2^

Secondly, we found an unexpected non-monotonic dependence of the transport dynamics with respect to the volume fraction. Counterintuitively, the dynamics in the labyrinthine phase is slower than in the tetragonal phase, despite its lower volume fraction and having an open structure which is only ordered in the *z*-direction. Moreover, the electric field effects the dynamics in opposite ways, depending on the phase of the system. The dynamics decrease with the electric field in the labyrinthine phase while accelerating for the other phases, which enhances the non-monotonicity raised above. This is reminiscent of recent work with active glasses where the state point can affect dramatically the dynamical behaviour.^27,64^ Keeping in mind that the electric field increases both the activity and the dipolar binding, our result suggests that one effect dominates to the other depending on the phase. This is all the more surprising given that the dipolar interaction is the predominant energy scale compared to the activity whatever the field strength. We believe that to gain an understanding of this behaviour, an appropriate strategy would be to perform a computer simulation study, to reproduce this phenomenon. We leave this as an open challenge for the future.

The 3D active crystals which form at higher volume fraction bear some similarity to those in the passive system, body-centred tetragonal at slightly lower volume fraction and then hexagonal at higher volume fraction. However the dynamical behaviour of active crystals is predicted to exhibit new kinds of excitations, and this would be intriguing to probe in the future.^65^

## 5 Conclusions

In conclusion, we have explored the phase behaviour and self-assembly of a 3D suspension of active colloids using particle-resolved experiments. Our observations have been carried out using a confocal microscope, providing access to microscopic details in space and in time. The competition between dipolar interactions and activity gives rise to a complex and rich behaviour, ranging from an isolated aggregate phase and an active dense disordered phase. In particular, with respect to the passive parent system,^43^ we have uncovered that the sheet phase is stabilised by activity. This could allow one to consider these sheets as a well-controlled model experimental system to study biological membrane fluctuations.^66^ These sheets can then undergo percolation in the *xy* plane to form a rather unusual labyrinthine phase, where particles form a structure reminiscent of a 2D network extended in the third dimension, but where activity leads the particles to jump from one branch to an another, and diffuse in the percolating network. It would be interesting to investigate how this structure responds to and whether or not it can sustain a mechanical stress like a colloidal gel. In closing we note that this work also opens new possibilities to study active matter in 3D at high density, where we can enquire how activity can affect phenomena such as percolation crystallisation and glass transition^27,64,67^ and phenomena such as “bubbly” phase separation.^68^ Finally, we note that we have only focussed on one frequency of the applied field. Given the behaviour of this class of Janus particles in 2D,^40^ one can expect more exotic behaviour by going to a higher frequency.

## Conflicts of interest

There are no conflicts to declare.

## Supplementary Material

SM-021-D5SM00182J-s001

SM-021-D5SM00182J-s002

SM-021-D5SM00182J-s003

SM-021-D5SM00182J-s004

SM-021-D5SM00182J-s005

SM-021-D5SM00182J-s006

SM-021-D5SM00182J-s007

## Data Availability

Data for this article, including representative microscopy images compiled into movies are available at Zenodo at DOI: https://doi.org/10.5281/zenodo.14894530.

## References

[cit1] Evans R., Frenkel D., Dijkstra M. (2019). Phys. Today.

[cit2] Royall C. P., Charbonneau P., Dijkstra M., Russo J., Smallenburg F., Speck T., Valeriani C. (2024). Rev. Mod. Phys..

[cit3] Ivlev A. , Löwen H., Morfill G. E. and Royall C. P., Complex Plasmas and Colloidal Dispersions: Particle-resolved Studies of Classical Liquids and Solids, World Scientific Publishing Co., Singapore, 2012

[cit4] Bishop K. J. M., Biswal S. B., Bharti B. (2023). Annu. Rev. Chem. Biomol. Eng..

[cit5] Needleman D., Dogic Z. (2017). Nat. Rev. Mater..

[cit6] Aubret A., Ramananarivo S., Palacci J. (2017). Curr. Opin. Colloid Interface Sci..

[cit7] Hagan M. F., Baskaran A. (2016). Curr. Opin. Cell Biol..

[cit8] Marchetti M. C., Joanny J.-F., Ramaswamy S., Liverpool T. B., Prost J., Rao M., Aditi Simha R. (2013). Rev. Mod. Phys..

[cit9] Bowick M. J., Fakhri N., Marchetti M. C., Ramaswamy S. (2022). Phys. Rev. X.

[cit10] Schwarz-Linek J., Valeriani C., Cacciuto A., Cates M. E., Marenduzzo D., Morozov A. N., Poon W. C. K. (2012). Proc. Natl. Acad. Sci. U. S. A..

[cit11] Czajkowski M., Bi D., Manning M. L., Marchetti M. C. (2018). Soft Matter.

[cit12] Petrolli V., Le Goff M., Tadrous M., Martens K., Allier C., Mandula O., Hervé L., Henkes S., Sknepnek R., Boudou T., Cappello G., Balland M. (2019). Phys. Rev. Lett..

[cit13] Sanchez T., Chen D. T. N., DeCamp S. J., Heymann M., Dogic Z. (2012). Nature.

[cit14] Guillamat P., Ignés-Mullol J., Sagués F. (2017). Nat. Commun..

[cit15] Attanasi A., Cavagna A., Del Castello L., Giardina I., Melillo S., Parisi L., Pohl O., Rossaro B., Shen E., Silvestri E., Viale M. (2014). PLoS Comput. Biol..

[cit16] Cavagna A., Giardina I. (2014). Annu. Rev. Condens. Matter Phys..

[cit17] Gasser U. (2009). J. Phys.: Condens. Matter.

[cit18] Mallory S. A., Valeriani C., Cacciuto A. (2018). Annu. Rev. Phys. Chem..

[cit19] Bechinger C., Di Leonardo R., Löwen H., Reichhardt C., Volpe G., Abd Volpe G. (2016). Rev. Mod. Phys..

[cit20] Al Harraq A., Bello M., Bharti B. (2022). Curr. Opin. Colloid Interface Sci..

[cit21] Ni R., Cohen Stuart M. A., Dijkstra M., Bolhuis P. G. (2014). Soft Matter.

[cit22] Palacci J., Sacanna S., Steinberg A. P., Pine D. J., Chaikin P. M. (2013). Science.

[cit23] Buttinoni I., Bialké J., Kümmel F., Löwen H., Bechinger C., Speck T. (2013). Phys. Rev. Lett..

[cit24] Cates M. E., Tailleur J. (2015). Annu. Rev. Condens. Matter Phys..

[cit25] Bricard A., Caussin J.-B., Desreumaux N., Dauchot D., Bartolo O. (2013). Nature.

[cit26] Aubret A., Youssef M., Sacanna S., Palacci J. (2018). Nat. Phys..

[cit27] Klongvessa N., Ginot F., Ybert C., Cottin-Bizonne C., Leocmach M. (2019). Phys. Rev. Lett..

[cit28] Fruchart M., Hanai R., Littlewood P., Vitelli V. (2021). Nature.

[cit29] Zhang J., Alert R., Yan J., Wingreen N. S., Granick S. (2021). Nat. Phys..

[cit30] Bililign E. S., Balboa Usabiaga F., Ganan Y. A., Poncet A., Soni V., Magkiriadou S., Shelley M. J., Bartolo D., Irvine W. T. M. (2022). Nat. Phys..

[cit31] Mauleon-Amieva A., Mosayebi M., Hallett J. E., Turci F., Liverpool T. B., van Duijneveldt J. S., Royall C. P. (2020). Phys. Rev. E.

[cit32] Stenhammar J., Marenduzzo D., Allen R. J., Cates M. E. (2014). Soft Matter.

[cit33] Omar A. K., Klymko K., GrandPre T., Geissler P. (2021). Phys. Rev. Lett..

[cit34] Turci F., Wilding N. B. (2021). Phys. Rev. Lett..

[cit35] Moore F. J., Russo J., Liverpool T. B., Royall C. P. (2023). J. Chem. Phys..

[cit36] Moore F. J., Royall C. P., Liverpool T. B., Russo J. (2021). Eur. Phys. J. E.

[cit37] Squires T. M., Brazant M. Z. (2006). J. Fluid Mech..

[cit38] Gangwal S., Cayre O. J., Bazant M. Z., Velev O. D. (2008). Phys. Rev. Lett..

[cit39] Nishiguchi D., Sano M. (2015). Phys. Rev. E: Stat., Nonlinear, Soft Matter Phys..

[cit40] Yan J., Han M., Zhang J., Xu C., Luijten E., Granick S. (2016). Nat. Mater..

[cit41] Zhang J., Yan J., Granick S. (2016). Angew. Chem., Int. Ed..

[cit42] Prymidis V., Sielcken H., Filion L. (2015). Soft Matter.

[cit43] Yethiraj A., van Blaaderen A. (2003). Nature.

[cit44] Hynninen A. P., Dijkstra M. (2005). Phys. Rev. Lett..

[cit45] Colla P. S., Mohanty T., Nöjid S., Riede A., Schurtenberger P., Likos C. (2018). ACS Nano.

[cit46] Cates M. E., Tailleur J. (2013). EPL.

[cit47] Wysocki A., Winkler R. G., Gompper G. (2014). EPL.

[cit48] Winkler R. G., Wysocki A., Gompper G. (2015). Soft Matter.

[cit49] Dassanayake U., Fraden S., van Blaaderen A. (2000). J. Chem. Phys..

[cit50] Stoy R. D. (1994). J. Electrost..

[cit51] Graf C., Vossen D. L. J., Imhof A., van Blaaderen A. (2003). Langmuir.

[cit52] Chao X., Skipper K., Royall C. P., Henkes S., Liverpool T. B. (2025). Phys. Rev. Lett..

[cit53] Leocmach M., Tanaka H. (2013). Soft Matter.

[cit54] Blair D. and Dufresne E., https://site.physics.georgetown.edu/matlab/

[cit55] Vercauteren T., Pennec X., Perchant A., Ayache N. (2009). NeuroImage.

[cit56] Yethiraj A., Wouterse A., Groh B., van Blaaderen A. (2004). Phys. Rev. Lett..

[cit57] Bialké J., Speck T., Löwen H. (2012). Phys. Rev. Lett..

[cit58] Richard D., Löwen H., Speck T. (2016). Soft Matter.

[cit59] Semwal S., Clowe-Coish C., Saika-Voivod I., Yethiraj A. (2022). Phys. Rev. X.

[cit60] Wu X., Skipper K., Yang Y., Moore F. J., Meldrum F. C., Royall C. P. (2025). Soft Matter.

[cit61] Furukawa A., Tanaka H. (2010). Phys. Rev. Lett..

[cit62] Royall C. P., Eggers J., Furukawa A., Tanaka H. (2015). Phys. Rev. Lett..

[cit63] Imamura S., Sawaki K., Molina J. J., Turner M. S., Yamamoto R. (2023). Adv. Theory Simul..

[cit64] Berthier L., Charbonneau P., Coslovich D., Ninarello A., Ozawa M., Yaida S. (2017). Proc. Natl. Acad. Sci. U. S. A..

[cit65] Caprini L., Marini Bettolo Marconi U., Löwen H. (2023). Phys Rev. E.

[cit66] Turlier H., Betz T. (2019). Annu. Rev. Condens. Matter Phys..

[cit67] Van Der Meer B., Dijkstra M., Filion L. (2016). Soft Matter.

[cit68] Tjhung E., Nardini C., Cates M. E. (2018). Phys. Rev. X.

